# Exploratory Study of Cesarean Scar Healing After Interrupted Versus Continuous Sutures: Prospective Magnetic Resonance Imaging Assessment in Cynomolgus Monkeys

**DOI:** 10.1002/rmb2.12695

**Published:** 2025-11-18

**Authors:** Ayako Inatomi, Shunichiro Tsuji, Yuri Nobuta, Daisuke Katsura, Yuji Tanaka, Atsushi Yamada, Takashi Murakami

**Affiliations:** ^1^ Department of Obstetrics and Gynecology Shiga University of Medical Science Otsu Shiga Japan; ^2^ Advanced Medical Research and Development Division, Medical Innovation Research Center Shiga University of Medical Science Otsu Shiga Japan

**Keywords:** cesarean scar disorder, cesarean section, double‐layer continuous sutures, double‐layer interrupted sutures, residual myometrial thickness

## Abstract

**Purpose:**

This study evaluated the effects of double‐layer interrupted sutures (DIS) and double‐layer continuous sutures (DCS) on uterine blood flow and residual myometrial thickness (RMT) in cynomolgus monkeys after cesarean section (CS).

**Methods:**

In DIS (*n* = 8) and DCS (*n* = 8) groups, uterine blood flow was assessed at 6 months post‐CS using MRI by Ktrans. RMT was measured by T2‐weighted magnetic resonance imaging (MRI) at 6 months. Laparoscopic evaluations were performed at 2 and 6 months.

**Results:**

At 6 months, Ktrans was significantly higher in the DIS group 6. RMT at the suture site did not differ significantly between groups. Adhesions were observed in three DIS and two DCS animals. Nonadhesive DIS animals had significantly higher Ktrans and greater RMT at 6 months compared with adhesive DIS animals. Nonadhesive DIS exhibited significantly higher Ktrans and greater RMT at 6 months than nonadhesive DCS.

**Conclusion:**

While overall differences were limited, exploratory findings indicate that DIS demonstrated superior uterine blood flow compared with DCS. Nonadhesive DIS animals exhibited greater RMT than adhesive DIS animals, suggesting a potential benefit of adhesion prevention.

## Graphical abstract

This graphical summary illustrates a prospective study evaluating cesarean scar healing after double‐layer interrupted sutures (DIS) versus double‐layer continuous sutures (DCS) in cynomolgus monkeys. At 6 months, MRI and laparoscopy revealed that DIS better preserved uterine blood flow and residual myometrial thickness (RMT) in nonadhesive cases.

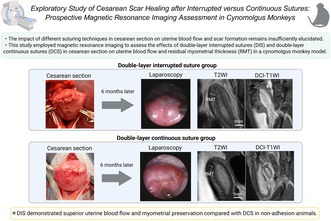

## 1 Introduction

Cesarean section (CS) is a critical obstetric procedure that protects the lives of mothers and their children. However, this often leads to myometrial thinning, which increases the risk of uterine rupture in subsequent pregnancies. Even in the absence of pregnancy, myometrial thinning can result in cesarean scar defects of the uterine isthmus, leading to postmenstrual spotting, dysmenorrhea, cesarean scar disorder (CSDi), or secondary infertility, significantly impairing the quality of life of postpartum women [1]. Therefore, addressing myometrial thinning during CS is crucial.

Preventive measures against myometrial thinning have been extensively studied. Double‐layer sutures are more effective than single‐layer sutures, whereas unlocked sutures outperform locked sutures [2, 3, 4, 5]. A recent randomized controlled trial comparing double‐layer continuous sutures (DCS) and double‐layer interrupted sutures (DIS) reported that the incidence of severe cesarean scar defects was significantly lower in the DIS group than in the DCS group [6]. However, the underlying mechanisms responsible for this difference remain unclear.

Emerging evidence has highlighted the critical role of adequate angiogenesis in uterine healing. In a rat model of partial uterine excision, interventions that promote angiogenesis restore full‐thickness repair and recover both the structure and function of the uterus [7, 8]. These findings suggest that the maintenance of uterine perfusion is essential for effective wound healing. Moreover, adhesions at the incision site can mechanically hinder tissue remodeling and reduce local blood supply [9]. Even with adhesion‐prevention materials like INTERCEED (Ethicon, Somerville, NJ, USA), adhesions may still occur, and their impact on uterine scar healing is not fully elucidated.

However, the evaluation of uterine blood flow using angiographic contrast agents in pregnant women after CS raises ethical concerns. Moreover, rodent models used in suturing studies exhibit substantial anatomical differences from humans, making them inappropriate for CS research [10].

A comparative study of DIS and DCS was conducted by performing CS on cynomolgus monkeys. Postoperative evaluations included laparoscopic examinations of the uterine suture site and periodic magnetic resonance imaging (MRI) using dynamic contrast‐enhanced MRI (DCE‐MRI) and T2 sequences. DCE‐MRI facilitated the quantitative assessment of tissue perfusion conditions around the suture site by deriving kinetic parameters such as the volume transfer constant between the plasma and extracellular extravascular space (EES) (Ktrans) and EES volume (Ve). T2 sequences were used to assess the residual myometrial thickness (RMT).

Therefore, this study prospectively evaluated the effects of DIS and DCS on uterine blood flow and RMT using MRI in a nonhuman primate CS model. Exploratory subgroup analyses were additionally conducted to examine the potential role of adhesions in modulating healing outcomes.

## 2 Materials and Methods

### 2.1 Animals

Sixteen cynomolgus monkeys ( *Macaca fascicularis* ) from Cambodia (*n* = 1), Vietnam (*n* = 11), and China (*n* = 4) were housed at the Center for Animal Life Science, Shiga University of Medical Science, from September 2022 to November 2024. As all monkeys were imported, their pregnancy and delivery histories were unknown. The monkeys were confirmed to be free of the herpes B virus, hepatitis E virus, *Mycobacterium tuberculosis* , *Shigella* spp., *Salmonella* spp., and enterohemorrhagic *Escherichia coli* . The animal housing conditions were maintained at a temperature of 25°C ± 2°C, humidity of 50% ± 5%, and a 12 h light–dark cycle. Animals were housed individually in separate cages with *ad libitum* access to food and water.

### 2.2 CS Procedure

Pregnant cynomolgus monkeys at 58–120 embryonic days conceived through in vitro fertilization at the Center for Animal Life Science, Shiga University of Medical Science, underwent CS under general anesthesia. Ketamine hydrochloride (5 mg/kg) and xylazine hydrochloride (1 mg/kg) were administered intramuscularly, followed by endotracheal intubation. Anesthesia was maintained with inhalational isoflurane at concentrations ranging from 0.5% to 2.0%.

CS involved a transverse uterine incision for fetal and placental delivery, followed by suturing of the uterine myometrium using 4–0 Vicryl (Ethicon, Somerville, NJ, USA). The monkeys were randomized into two groups: DIS (*n* = 8) and DCS (*n* = 8). In both groups, the first layer of suturing involved closure of approximately two‐thirds of the myometrium, including a thin portion of the decidua. The second‐layer needles were inserted between the sutures of the first layer. To prevent adhesions, INTERCEED (Ethicon, Somerville, NJ, USA) (96.5 cm^2^) was applied to the uterine incision site prior to abdominal closure. All procedures were performed by an obstetrician–gynecologist (A.I.).

### 2.3 Laparoscopic Examination

A single investigator performed minimally invasive laparoscopic examinations to minimize animal discomfort. Anesthesia was induced using ketamine (5 mg/kg) and xylazine (1 mg/kg). A 3‐mm trocar was inserted along the midline, caudal to the umbilicus, to introduce a laparoscope (LA‐6500; Machida Endoscope, Chiba, Japan). A second trocar was placed in the right lateral abdomen to insert a surgical probe, and approximately 200 mL of filtered air was insufflated into the abdominal cavity to create an operative field.

The laparoscope was connected to a charge‐coupled device camera, and images were displayed and recorded on a hard‐disk system (Shimizu Laboratory Supply Co., Kyoto, Japan). The observation lasted approximately 3–5 min, and the entire procedure was completed within 10 min. Laparoscopic examinations were performed at 2 and 6 months postoperatively in both groups to evaluate the uterine suture site for macroscopic changes such as color change, depression, and adhesion formation.

### 2.4 MRI Examination

Ketamine hydrochloride (5 mg/kg) and xylazine hydrochloride (1 mg/kg) were administered intramuscularly to induce anesthesia, which was maintained with 0.5%–1.5% isoflurane. Spontaneous respiration was monitored using a video camera during imaging, and oxygen saturation (SpO2) was continuously monitored using an SpO2 sensor. Oxygen supplementation ensured that SpO2 levels remained ≥ 96%.

MRI scans were conducted at postoperative intervals of 6 months using a 3 T MRI scanner (MAGNETOM Verio; Siemens Healthcare, Erlangen, Germany). The imaging protocols included T1‐weighted imaging (T1WI), T2‐weighted imaging (T2WI), T2*‐weighted imaging (T2*WI), and DCE‐MRI sequences for tissue perfusion analysis (Figure 3A). A specialized body matrix coil was used to enhance image quality. For contrast‐enhanced imaging, a cyclic nonionic MRI contrast agent (gadoterate meglumine injection, 38%; GE Healthcare Pharma, Tokyo, Japan) was administered intravenously at a dose rate of 1 mL/kg and an injection rate of 2.0 mL/s.

The uterine suture site was identified by an obstetrician‐gynecologist (A.I.) using intraoperative findings, laparoscopic examination, and T1WI and T2WI. The RMT at the suture site was measured on T2WI at 6 months postoperatively. The uterine wall exhibits a characteristic three‐layered structure comprising a hyperintense endometrium, a hypointense junctional zone (JZ), and a relatively hyperintense outer myometrium. The RMT was measured from the outer border of the JZ to the inner surface of the serosa (Figure 3B).

### 2.5 MRI Acquisition Parameters

All MRI sequences were acquired using a generalized autocalibrating partially parallel acquisition technique with an acceleration factor of 2. The scan parameters were as follows:

- T1WI: T1‐weighted turbo spin‐echo (TSE) BLADE: repetition time (TR) = 550 ms, echo time (TE) = 10 ms, field of view (FOV) = 150 × 150 mm, matrix size = 179 × 256, slice thickness = 2.5 mm, average = 2, flip angle = 120.
- T2WI: T2‐weighted TSE BLADE: TR, 4500 ms; TE, 93 ms; FOV, 150 mm × 150 mm; matrix size, 224 × 320; slice thickness = 2.5 mm, averages = 3, flip angle, 124.
- T2*: T2*‐weighted gradient echo with a fast low‐angle shot sequence: TR = 600 ms, TE = 9.84 ms, FOV = 150 × 150 mm, matrix size = 179 × 256, slice thickness = 2.5 mm, average = 2, flip angle = 20.
- DCE‐MRI: Corrected T1‐weighted volumetric interpolated breath‐hold examination: TR = 5.30 ms, TE = 1.94 ms, FOV = 120 mm × 105 mm, matrix size = 94 × 160, slice thickness = 1.5 mm, averages = 1, flip angle = 15°, total of 40 scan periods (7.5 s per period).

### 2.6 DCE‐MRI Postprocessing Analysis

The acquired imaging data were processed using the postprocessing application Tissue 4D (Siemens Healthcare). The preprocessing steps included motion correction and image matching. The region of interest (ROI) of the uterine suture site was delineated using intraoperative findings, laparoscopic examinations, and T1WI and T2WI data (Figure 4). This procedure was performed by an obstetrician/gynecologist (A.I.). Multiple kinetic parameters, such as Ktrans and Ve, within the ROI were calculated and measured using Tofts' two‐compartment pharmacokinetic model.

### 2.7 Sample Size Determination

Based on a two‐sample t‐test for two independent groups, the minimum sample size required to detect differences in Ktrans was calculated using the formula: $n\geq 2\times {\left(\frac{\mathrm{SD}\;}{\Delta }\right)}^{2}\times {\left(Z_{\alpha /2}+Z_{\beta }\;\right)}^{2}$. Δ represents the mean difference between the two groups, and SD represents the average of the standard deviations from each group. Assuming a significance level of α = 0.05 ($Z_{\alpha /2}$ = 1.96), a power of 80% ($Z_{\beta }$ = 0.84), a standard deviation (SD) of 0.026525, and a detectable difference (Δ) of 0.03742, the required sample size was estimated as 16 animals (*n* = 8 per group).

### 2.8 Statistical Analyses

Numerical data are expressed as mean ± standard error of the mean (SEM). Normality was assessed using the Shapiro–Wilk test. Between‐group differences in continuous and categorical variables were evaluated using two‐tailed unpaired t‐tests and the chi‐square test, respectively. Pearson's correlation analysis was used to assess the relationship between the RMT and Ktrans. Statistical significance was defined as *p* < 0.05. All analyses were conducted using GraphPad Prism version 10.0.0 for Windows (GraphPad Software, Boston, MA, USA).

## 3 Results

All 16 cynomolgus monkey dams underwent CS. The mean age of the dams was 8.5 ± 0.8 years in the DIS group and 8.4 ± 0.8 years in the DCS group (*p* = 0.89). The mean body weight was 3.76 ± 0.21 kg in the DIS group and 3.32 ± 0.14 kg in the DCS group (*p* = 0.11).

### 3.1 Laparoscopic Examination

**FIGURE 1:**
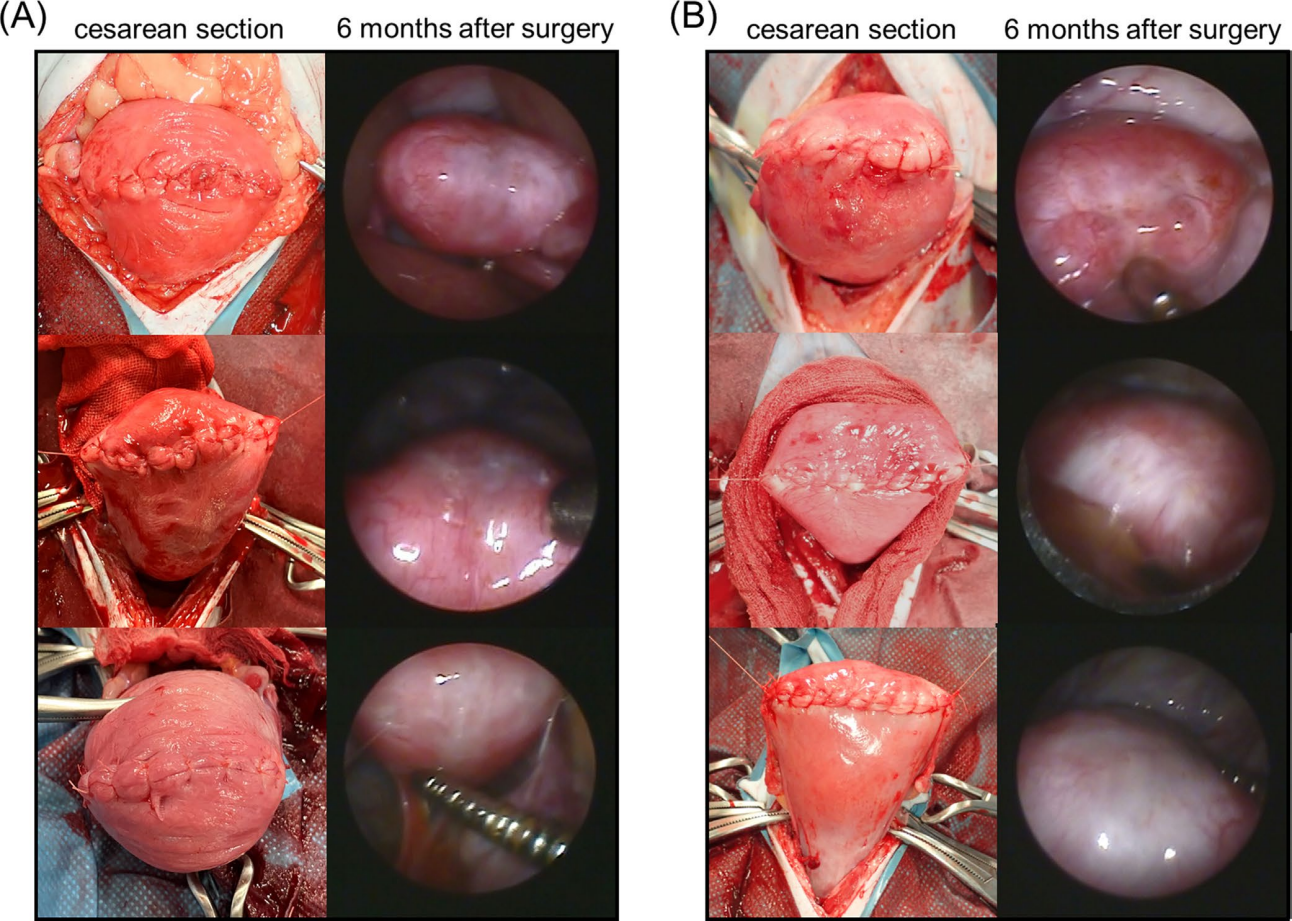
Intraoperative and 6 month postoperative laparoscopic findings of the uterine incision site following two different suturing techniques in cynomolgus monkeys. (A) Three animals from the double‐layer interrupted suturing group. (B) Three animals from the double‐layer continuous suturing group. For both panels, the left‐side images were obtained intraoperatively during the cesarean section, whereas the right‐side images were captured 6 months postoperatively via laparoscopy.

**FIGURE 2:**
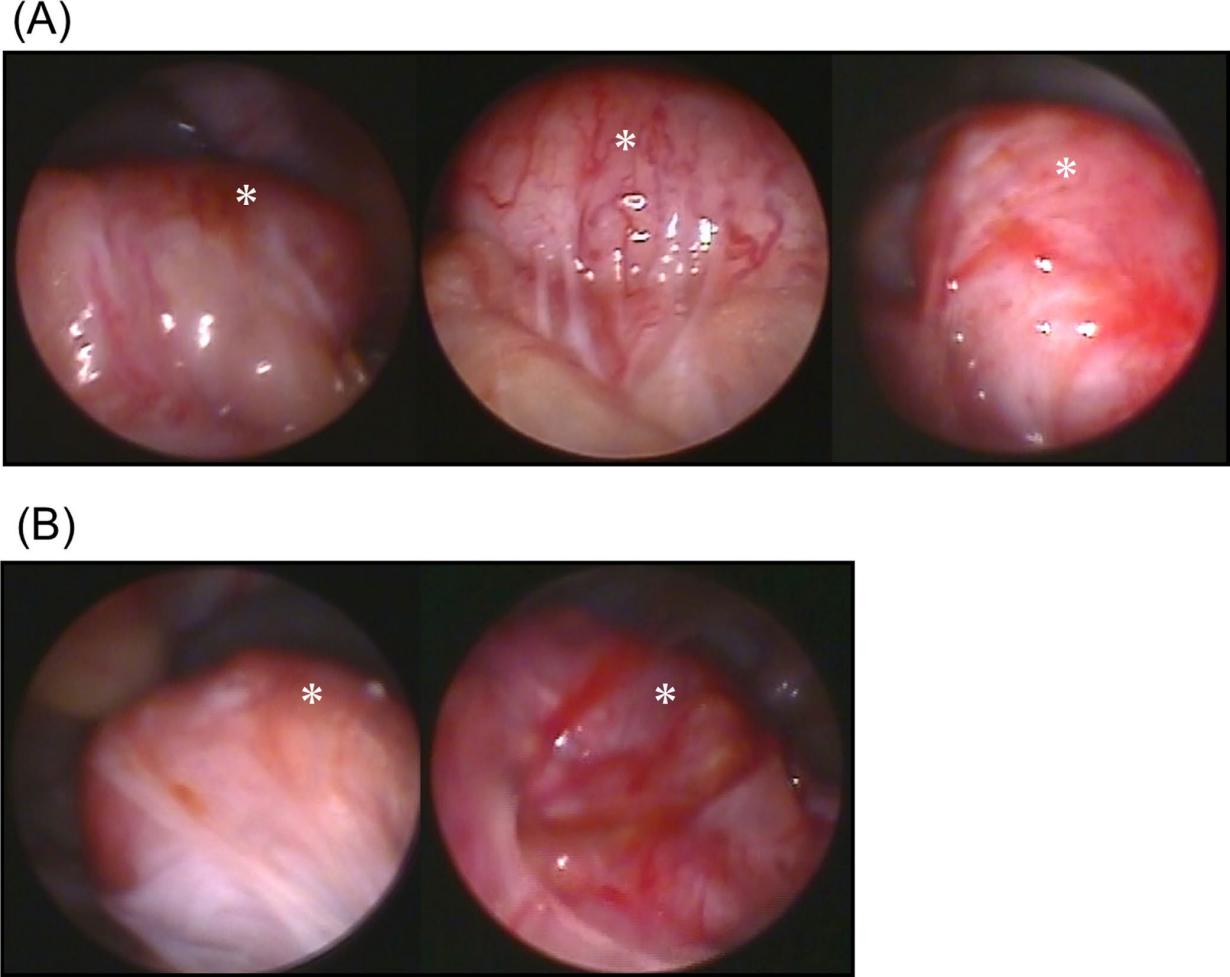
Laparoscopic findings of adhesion formation at the uterine incision site 2 months postoperatively. (A) Three animals from the double‐layer interrupted suturing group. (B) Two animals from the double‐layer continuous suturing group. The white asterisks indicate the uterus.

Figure 1 depicts images of the uterus taken during CS and at 6 months postoperatively, obtained via laparoscopic examination. Two months postoperatively, five animals exhibited strong omental adhesions at the uterine incision site (DIS group, *n* = 3; DCS group, *n* = 2; *p* = 0.50) (Figure 2).

At 6 months postoperatively, laparoscopic examinations of the uterine incision site were performed in animals without adhesions. Uterine scar whitening was observed in one (20%) of five animals in the nonadhesive DIS group and in five (83.3%) of six animals in the nonadhesive DCS group (*p* = 0.07). Depression at the scar site was observed in none (0%) of the five animals in the nonadhesive DIS group and in one animal (16.7%) of the six animals in the nonadhesive DCS group (*p* = 0.55).

### 3.2 MRI Examination

**FIGURE 3:**
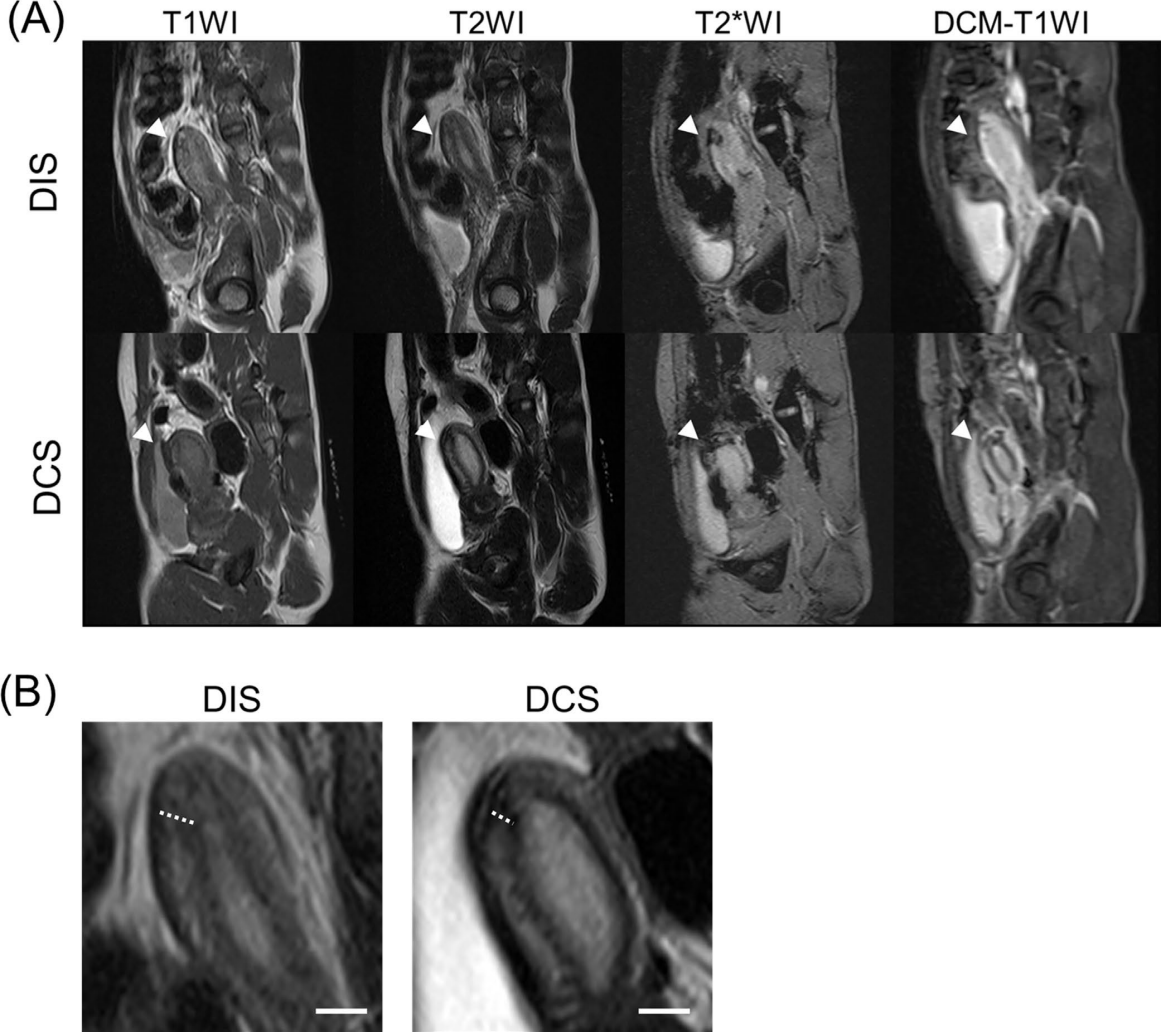
Sagittal magnetic resonance imaging findings of the uterine incision site at 6 months postoperatively in cynomolgus monkeys from the double‐layer interrupted and double‐layer continuous suturing groups. (A) Sagittal images include T1WI, T2WI, T2*WI, and DCE‐T1WI. Upper row, DIS group; lower row, DCS group. White arrowheads indicate the uterine incision site. (B) Measurement of RMT on T2WI. The uterine wall exhibits a characteristic three‐layered structure consisting of a hyperintense endometrium, a hypointense JZ, and a relatively hyperintense outer myometrium. The RMT was measured from the outer border of the JZ to the inner surface of the serosa. Left, DIS group; right, DCS group. The dotted lines indicate the measurement locations. Scale bars = 5 mm. T1WI, T1‐weighted imaging; T2WI, T2‐weighted imaging; T2*WI, T2*‐weighted imaging; DCE‐T1WI, dynamic contrast‐enhanced T1‐weighted imaging; DIS, double‐layer interrupted suturing; DCS, double‐layer continuous suturing; RMT, residual myometrial thickness; JZ, junctional zone.

**FIGURE 4:**
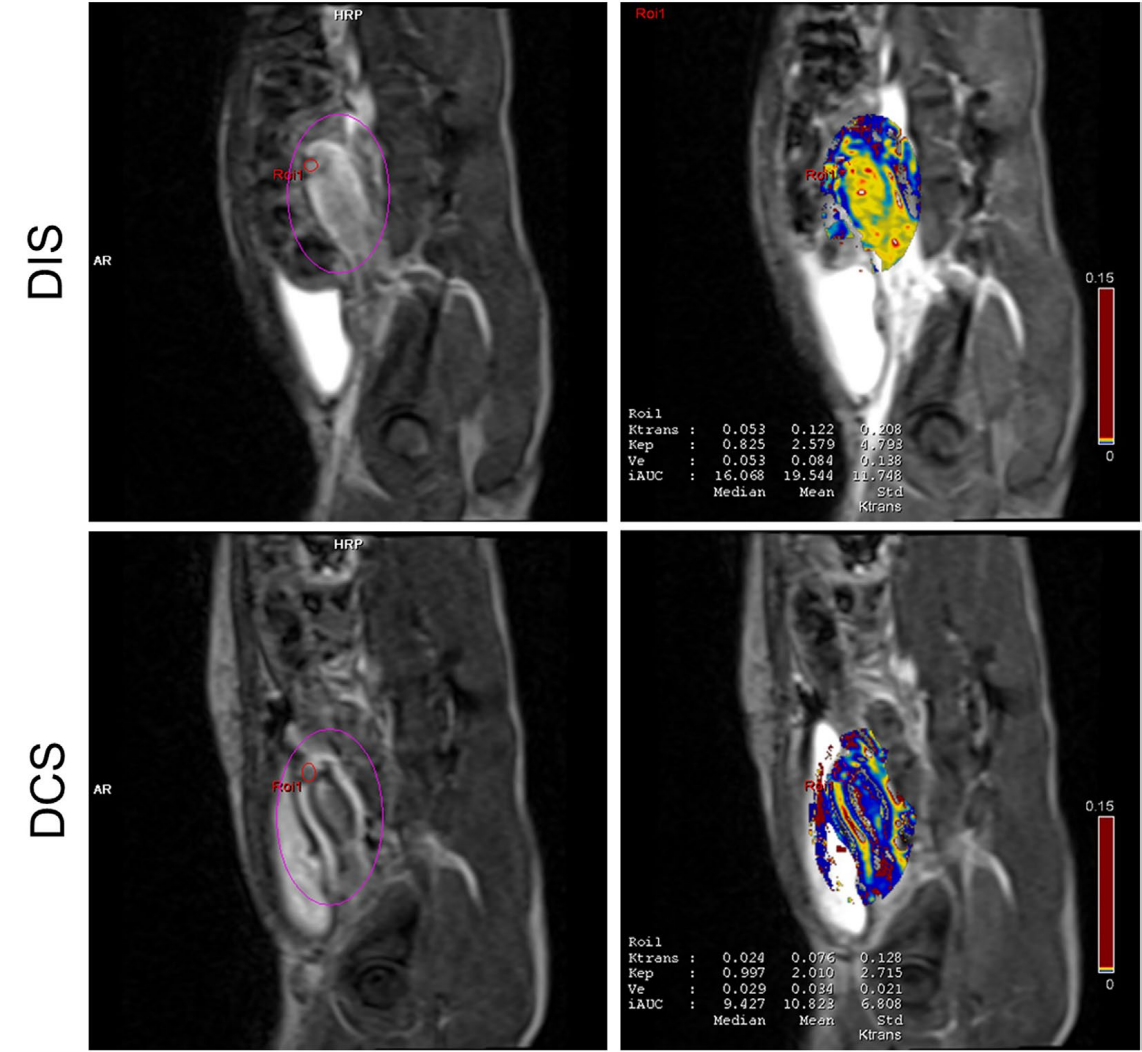
Tissue 4D analysis of uterine blood flow 6 months postoperatively. Left, Region‐of‐interest selection images; right, corresponding blood flow analysis results; top, DIS group; bottom, DCS group. DIS, double‐layer interrupted suturing; DCS, double‐layer continuous suturing.

Figures 3 and 4 present the T1WI, T2WI, T2*WI, DCE‐MRI, and Tissue 4D images at 6 months postoperatively of the DIS and DCS groups. As shown in Figure 3, T2WI clearly demonstrated the layered uterine wall structure, allowing reliable measurement of the RMT at the incision site. As shown in Figure 4, Tissue 4D analysis allowed visualization of regional blood flow at the uterine incision site. The DIS group exhibited greater enhancement at the scar site, suggesting improved perfusion compared with that in the DCS group.

**FIGURE 5:**
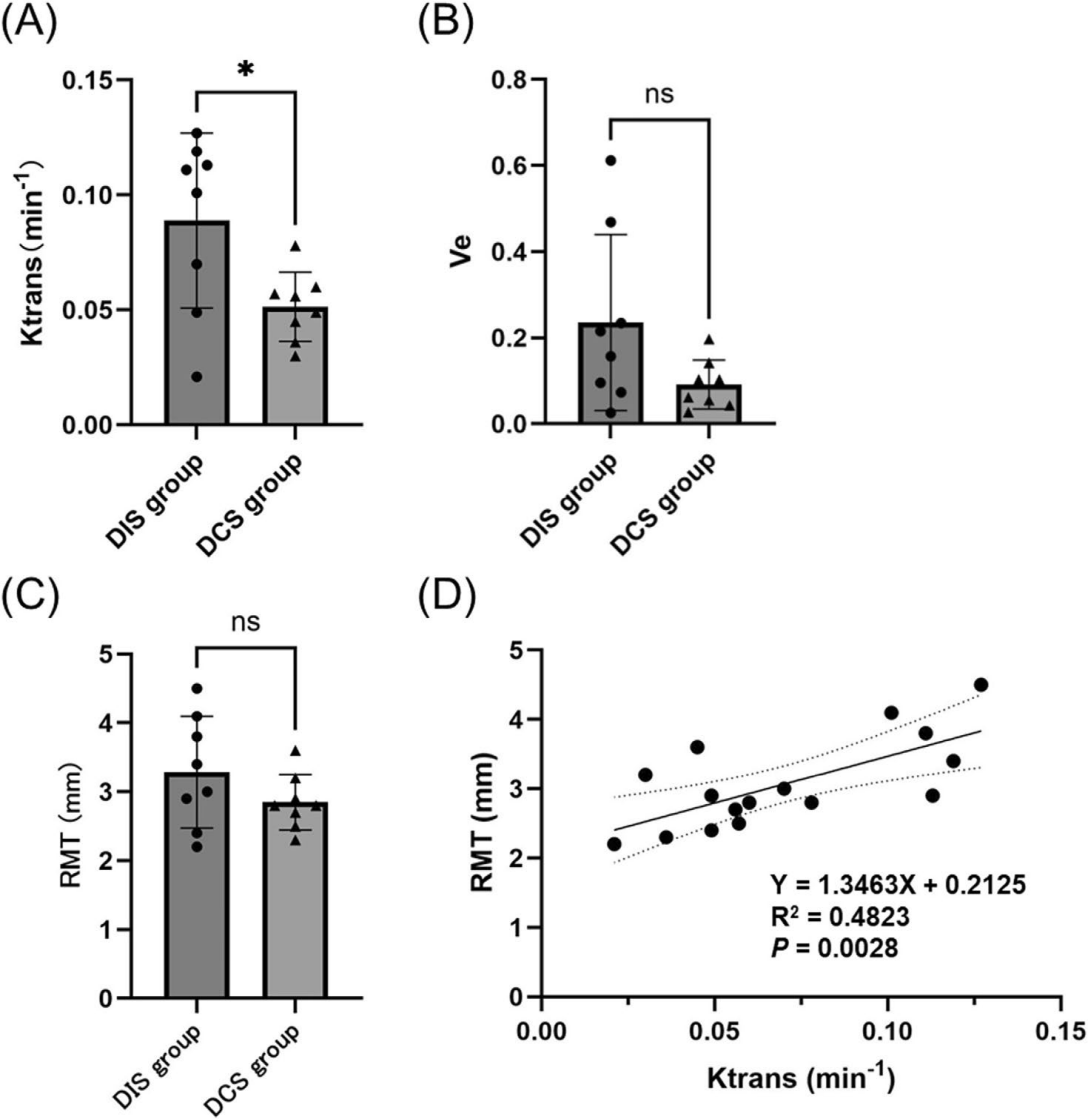
Kinetic parameters and RMT at 6 months postoperatively in 16 cynomolgus monkeys. (A) Ktrans values at 6 months postoperatively. At 6 months postoperatively, the Ktrans value was significantly higher in the DIS group (*n* = 8) than in the DCS group (*n* = 8) (*p* < 0.05). (B) Ve values at 6 months postoperatively. No significant differences were observed between the group. (C) RMT at 6 months postoperatively. No significant differences were observed between the DIS and DCS groups. (D) Correlation between the RMT and Ktrans value at 6 months postoperatively. A significant positive correlation was observed (R^2^ = 0.4823, *p* = 0.0028). Left bar, DIS group; right bar, DCS group; ● DIS group; ▲ DCS group. Statistical analyses were performed using a t‐test. *p < 0.05. ns, not significant; RMT, residual myometrial thickness; Ktrans, volume transfer constant between the plasma and extracellular extravascular space; Ve, extracellular extravascular space volume; DIS, double‐layer interrupted suturing; DCS, double‐layer continuous suturing.

At 6 months postoperatively, the Ktrans values for the suture site assessed using Tissue 4D were significantly higher in the DIS group than in the DCS group (Cohen's d = 1.297, 95% Confidence Interval −0.06851 to −0.006487, *p* < 0.05) (Figure 5A). At 6 months postoperatively, the Ve values showed no significant differences between the suturing methods (Figure 5B). The RMT at the suture site measured 6 months postoperatively revealed no significant difference between the groups (Figure 5C). Furthermore, a moderate positive correlation was observed between Ktrans values and the RMT at the suture site (R^2^ = 0.4823, *p* < 0.01) (Figure 5D), suggesting that increased perfusion may be associated with greater myometrial preservation.

**FIGURE 6:**
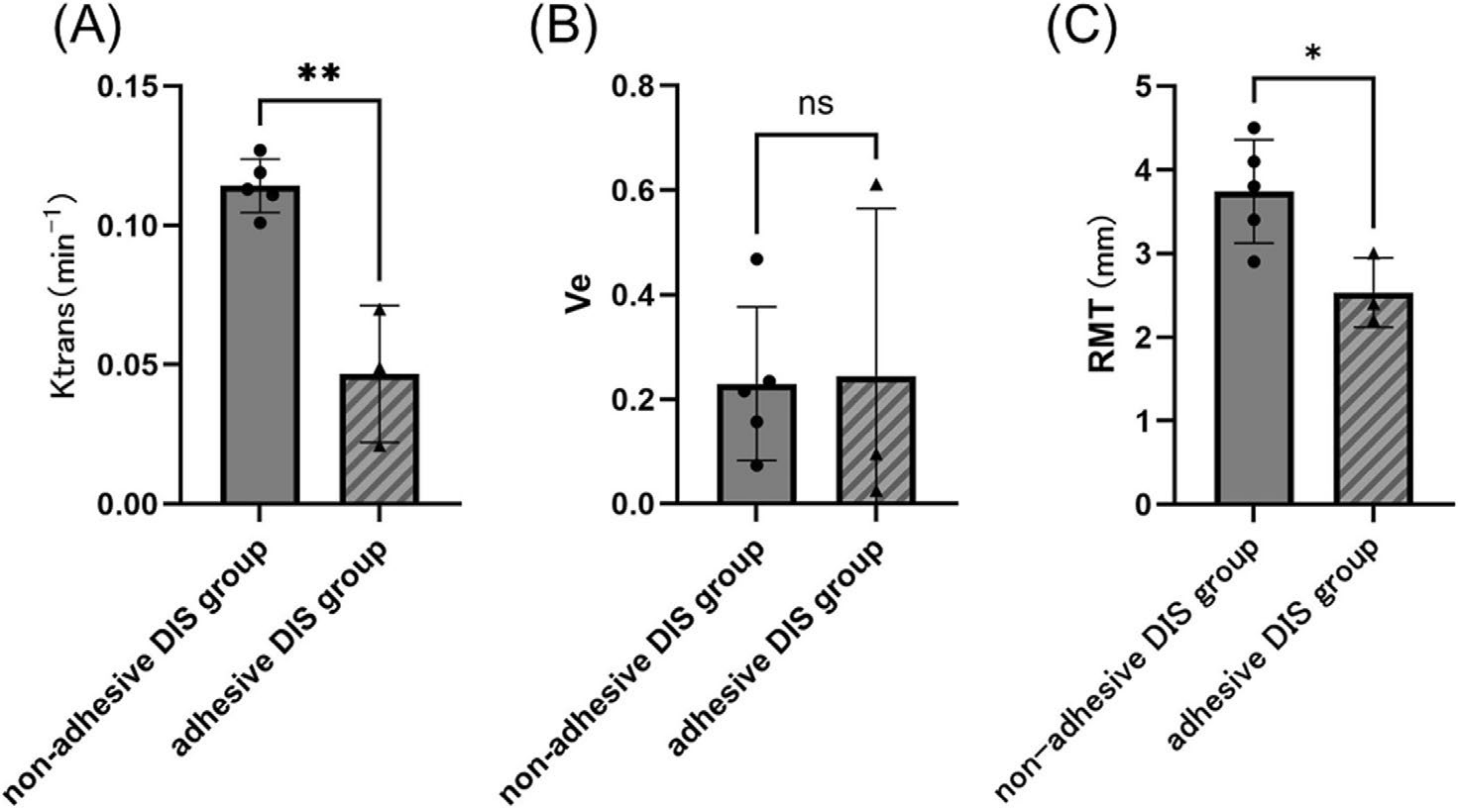
Kinetic parameters and RMT at 6 months postoperatively in eight cynomolgus monkeys with interrupted suturing. (A) Ktrans values at 6 months postsurgery. The Ktrans values were significantly higher in the nonadhesive DIS group (*n* = 5) than in the adhesive DIS group (*n* = 3) (*p* < 0.01). (B) Ve values at 6 months postsurgery. No significant differences were observed between the two groups. (C) RMT at 6 months postoperatively, showing a significantly greater RMT in the nonadhesive DIS group than in the adhesive DIS group (*p* < 0.05). Statistical analyses were performed using a t‐test. *p < 0.05, **p < 0.01. Nonshaded bars, nonadhesive group; shaded bars, adhesive group; ns, not significant; RMT, residual myometrial thickness; Ktrans, volume transfer constant between the plasma and extracellular extravascular space; Ve, extracellular extravascular space volume.

Subgroup analysis was performed to compare the outcomes in the DIS group between the adhesive and nonadhesive groups. At 6 months postoperatively, the Ktrans values were significantly higher in the nonadhesive DIS group than in the adhesive DIS group (Cohen's d = 4.160, 95% Confidence Interval −0.09654 to −0.03852, *p* < 0.01) (Figure 6A). In contrast, the Ve values at 6 months postoperatively showed no significant differences between the two suturing methods (Figure 6B). At 6 months postoperatively, the RMT was significantly greater in the nonadhesive DIS group than in the adhesive DIS group (Cohen's d = 2.207, 95% Confidence Interval −2.207 to −0.2067, *p* < 0.05) (Figure 6C). However, given the small subgroup sizes (*n* = 3 vs. *n* = 5), this analysis should be considered exploratory, and the *p* value is presented for transparency. In the DCS group at 6 months postoperatively, comparison between adhesive (*n* = 2) and nonadhesive (*n* = 6) animals yielded *p* = 0.1395 for Ktrans and *p* = 0.4899 for Ve. Due to the very small number of adhesive animals, these results were reported but not interpreted further.

**FIGURE 7:**
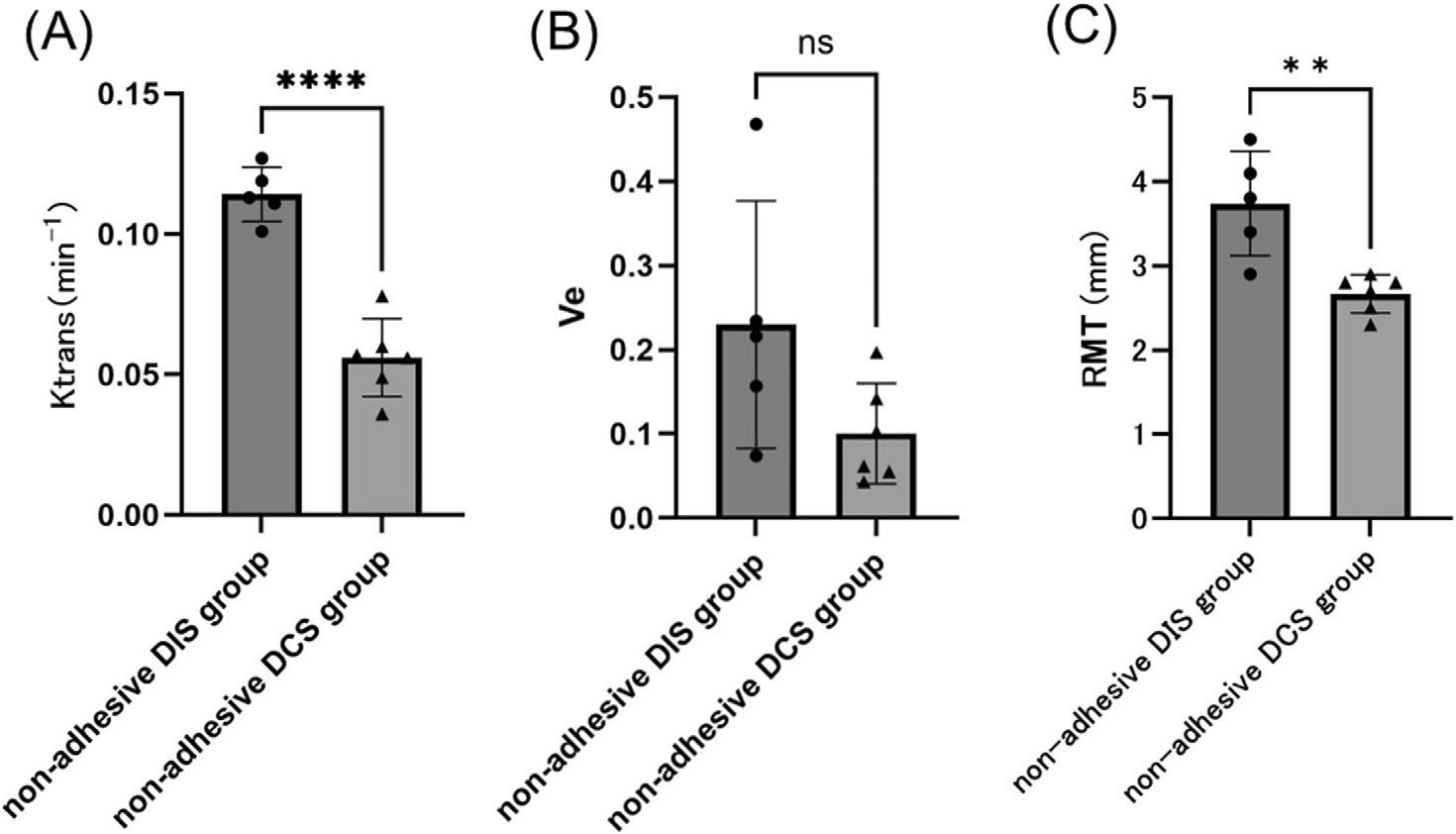
Kinetic parameters and RMT 6 months postoperatively in 11 cynomolgus monkeys without adhesions. (A) Ktrans values at months after surgery. The Ktrans values were significantly higher in the nonadhesive DIS group (*n* = 5) than in the nonadhesive DCS group (*n* = 6) (*p* < 0.0001). (B) Ve values at 6 months postsurgery. There were no significant differences between the two groups. (C) RMT at 6 months postoperatively, showing a significantly greater RMT in the nonadhesive DIS group than in the nonadhesive DCS group (*p* < 0.01). Statistical analyses were performed using a t‐test. **p < 0.01, ****p < 0.0001. Left bars, DIS group; right bars, DCS group; ● DIS group; ▲ DCS group; ns, not significant; RMT, residual myometrial thickness; Ktrans, volume transfer constant between the plasma and extracellular extravascular space; Ve, extracellular extravascular space volume.

As poor uterine blood flow was observed in monkeys with adhesions, an additional subgroup analysis was performed for animals without adhesions between the DIS and DCS groups. At 6 months postoperatively, the Ktrans values were significantly higher in the nonadhesive DIS group than in the nonadhesive DCS group (Cohen's d = 4.80, 95% Confidence Interval −0.07481 to −0.04159, *p* < 0.0001) (Figure 7A). In contrast, the Ve values at 6 months postoperatively showed no significant differences between the suturing methods or across time points (Figure 7B). The RMT at the suture site at 6 months postoperatively was significantly greater in the nonadhesive DIS group than in the nonadhesive DCS group (Cohen's d = 2.41, 95% Confidence Interval −1.683 to −0.4632, *p* < 0.01) (Figure 7C).

## 4 Discussion

This study demonstrated that, at 6 months postoperatively, no significant between‐group difference in RMT was detected. In contrast, DIS was more effective than DCS in maintaining blood flow at the uterine incision site. Subgroup analysis of animals without adhesions confirmed the superiority of interrupted sutures in preserving the uterine blood flow and RMT, as the Ktrans values were consistently higher and the RMT remained thicker in the nonadhesive DIS group than in the nonadhesive DCS group. This study reinforces the potential benefits of interrupted suturing in maintaining uterine blood flow and RMT. Additionally, adhesions at the uterine incision site are associated with reduced uterine blood flow and RMT in the DIS group. To our knowledge, this is the first study to objectively demonstrate an association between adhesions at the uterine scar site and uterine blood flow and RMT.

Current evidence indicates that interrupted suturing may better preserve myometrial thickness and lower the incidence of niche formation, defined as the development of a localized indentation at the uterine scar site. Studies have shown that continuous suturing increases the risk of niche formation compared to that with interrupted suturing [6], consistent with our findings. Niche formation is associated with intrauterine fluid retention, which increases infertility risk [11, 12, 13]. Therefore, the selection of an optimal suturing technique to prevent niche formation is crucial. Although our study did not directly measure niche formation, the finding that DIS prevented myometrial thinning in non‐adhesive animals may serve as an important consideration for the selection of suturing techniques for future CS procedures. In contrast, recent reports suggest that modern techniques, such as barbed sutures, effectively reduce the formation of niches even with continuous suturing [14, 15]. Therefore, future studies comparing traditional interrupted sutures with these contemporary methods are warranted to determine the optimal approach for uterine closure.

Furthermore, existing studies highlight the advantages of early surgical treatment of pelvic adhesions in endometriosis [16, 17, 18, 19, 20]. Based on these findings, surgical removal of pelvic adhesions may enhance uterine blood flow. Furthermore, reports suggest that post‐CS adhesions between the uterine scar and the abdominal wall may impair incision site healing and contribute to cesarean scar defect‐induced CSDi [9, 21]. The prevalence of adhesions during a second CS ranges from 12% to 46% and increases to 26%–75% during a third CS [22, 23]. During laparoscopic niche repair surgery for CSDi, fibrotic adhesions between the scar and the abdominal wall are frequently observed at the deepest point of the niche [9]. These adhesions may pull the scar tissue toward the abdominal wall, exerting a counteracting force that opposes normal uterine scar contraction, which is crucial for optimal myometrial re‐approximation and proper healing. The findings of this study, together with prior reports, suggest that adhesions at the uterine incision site may compromise uterine blood flow, impair scar healing, and contribute to CSDi. However, although statistical significance was observed between adhesive and nonadhesive DIS subgroups, the subgroup sizes were very small (*n* = 3 vs. *n* = 5). Therefore, these results should be regarded as exploratory and hypothesis‐generating rather than definitive. Furthermore, existing studies highlight the advantages of early surgical treatment of pelvic adhesions in endometriosis [16, 17, 18, 19, 20]. Based on these findings, surgical removal of pelvic adhesions may enhance uterine blood flow.

The findings of this study, together with prior reports, suggest that adhesions at the uterine incision site may compromise uterine blood flow, impair scar healing, and contribute to CSDi. However, although statistical significance was observed between adhesive and nonadhesive DIS subgroups, the subgroup sizes were very small (*n* = 3 vs. *n* = 5). Therefore, these results should be regarded as exploratory and hypothesis‐generating rather than definitive. In this study, intra‐abdominal adhesions were observed in five of 16 animals (31.3%) despite the application of INTERCEED to all uterine incisions. A sufficiently large sheet (96.5 cm^2^) was consistently applied to cover the incision, and the method of application was standardized across animals, making variability in surgical technique unlikely. One possible contributing factor is species‐specific postoperative behavior: unlike human patients who remain at rest, monkeys were ambulatory immediately after recovery from anesthesia, which may have displaced or destabilized the barrier material. Thus, the relatively high adhesion rate observed here may reflect both the unique postoperative activity of nonhuman primates and their potential predisposition to adhesion formation, rather than inconsistencies in adhesion‐prevention techniques.

No significant difference in the Ve values was observed between the two suturing methods, suggesting that the suturing techniques did not influence tissue cellularity. Ve represents the fractional volume of the EES affected by cell proliferation and necrosis. As cell density increases, the available extracellular space for contrast agent leakage decreases, resulting in a lower Ve value. In oncologic DCE‐MRI studies, Ve typically increases due to tumor necrosis and loss of cell–cell adhesion molecules, both of which expand the interstitial space [24]. Although fibrosis and other changes may have occurred in this study because of suturing, the examined tissue remained normal, with no evidence of necrosis or loss of cell adhesion molecules. Therefore, the Ve values were likely to remain unaffected.

To our knowledge, this is the first prospective observational study to investigate CS suturing techniques using a cynomolgus monkey model and analyze the uterine incision site using MRI. A key feature of this study was the use of DCE‐MRI to evaluate uterine blood flow quantitatively. Although studies on uterine blood flow using DCE‐MRI are limited, research in other medical fields using similar methodologies has demonstrated that Ktrans serves as a reliable indicator of tissue perfusion and healing [25, 26]. Furthermore, the cynomolgus monkey model is highly relevant for research on uterine suturing in humans, owing to its anatomical similarity to the human uterus. Conversely, rodents are less suitable for human reproductive surgery because they have bicornuate uteri, lack menstruation, and possess fallopian tubes that do not open into the peritoneal cavity. Monkeys possess anatomical structures of the uterus, fallopian tubes, and ovaries that are similar to those of humans, along with a menstrual cycle of approximately 28 days, making them highly relevant for translational research in human obstetrics and gynecology [10].

However, this study has some limitations. First, the uterine incision site in monkeys differs from that in humans because it is located near the fundus. In cynomolgus monkeys, the lower uterine segment is anatomically narrow, making the standard low transverse incision technically impossible during CS. Therefore, in this study, a transverse incision was made near the anterior aspect of the uterine fundus. Although fundal incisions are not commonly used for CS in humans, they are occasionally used in the presence of certain conditions, such as placenta previa. Despite the need for careful postoperative management, previous studies have shown that this type of incision facilitates adequate repair of the myometrium and allows future pregnancies [27]. While future studies should ideally replicate the low transverse incision commonly used for CS in humans, we consider the current model valid for the evaluation of uterine healing. Second, only blood flow parameters were evaluated using DCE‐MRI, with no accompanying histological analysis. Because histological analysis was not performed in this study, we were unable to evaluate tissue fibrosis or angiogenesis; thus, we could not corroborate the tissue perfusion findings obtained via Tissue 4D analysis. In future studies, we plan to incorporate histological evaluation through biopsy of the suture site to provide a more comprehensive assessment of uterine healing. Thirdly, the sample size estimation was based solely on Ktrans, a perfusion parameter derived from DCE‐MRI. Therefore, the statistical power to detect differences in other outcomes, such as RMT, may be limited. The findings related to secondary endpoints should be interpreted cautiously, and future studies with larger cohorts are required to validate these results across multiple parameters.

In conclusion, among cynomolgus monkeys that underwent CS, myometrial preservation and uterine blood flow were better with DIS than with DCS in animals without adhesions. The absence of adhesions at the uterine incision site may be associated with improved uterine healing. These findings suggest the potential benefit of preventing adhesions in terms of superior postoperative outcomes.

## Ethics Statement

This study was approved by the Shiga University of Medical Science Ethics Committee for Animal Experiments (approval no. 22–094) and adhered to the guidelines of the Japanese Physiological Society.

## Conflicts of Interest

The authors declare no conflicts of interest.

## Data Availability

The data that support the findings of this study are available from the corresponding author upon reasonable request.
